# Anti-Multipath Performance Improvement of an M-ary Position Phase Shift Keying Modulation System

**DOI:** 10.3390/s19081938

**Published:** 2019-04-25

**Authors:** Haiyuan Wang, Hongxian Tian

**Affiliations:** School of Electronic and Information Engineering Beijing Jiaotong University, Beijing 100044, China; 17120121@bjtu.edu.cn

**Keywords:** M-ary position phase shift keying, multipath, joint judgment

## Abstract

Low-Power Wide-Area Network (LPWAN) is the technology that the Internet-of-Things (IoT) uses in long-distance, wide-coverage scenarios. As one of the ultra-narrowband (UNB) modulation techniques, M-ary position phase shift keying (MPPSK) modulation can provide high coverage and high reliability for LPWAN. This paper proposes a multipath separation method based on MPPSK modulation, which aims to eliminate the influence of multipath on the main path without increasing the spectrum overhead and system complexity. Specifically, the modulation parameter of the system is adjusted according to the delay value, so that the phase jump of the multipath signal falls outside the phase jump of the main path symbol to achieve separation of the multipath from the main path. Moreover, a normalized symbol joint decision method is proposed in order to reduce the introduced noise while using multipath information for decisions. The simulation results indicate that the multipath separation conditions given in this paper can meet the requirements of multipath separation of MPPSK signals. Compared with the existing mainstream decision scheme, the normalized symbol joint decision improves the demodulation performance of the system.

## 1. Introduction

The Internet-of-Things is a new concept that is increasingly attracting the attention of researchers and industries [1]. It is expected that by 2020, there will be 20 billion communicating objects in the world [2]. In order to meet the demand of so many connectec devices, LPWAN that can realize long-distance transmission and reduce communication power consumption is proposed [3]. Ultra-narrowband modulation with high spectral efficiency as the physical layer technology of LPWAN and can meet the large coverage and high reliability requirements of LPWAN [2,4,5,6,7,8]. In order to achieve very high spectral efficiency without excessively reducing power efficiency, modulation of M-ary Position Phase Shift Keying (MPPSK) is proposed based on Extend Binary Phase Shift Keying (EBPSK) modulation [9,10,11]. As a multi-band form of EBPSK modulation, it is an efficient and flexible modulation method. The receiver can achieve demodulation by extracting tiny modulation information using a extremely narrow pass band filter called a digital impacting filter (DIF). In [12,13], the special mechanism of DIF is explained in detail. MPPSK can flexibly select modulation schemes, change bit rate, power efficiency and spectrum efficiency by changing modulation parameters. Compared with traditional modulation methods, MPPSK also has the advantages of high bandwidth efficiency and line spectrum elimination. Therefore, it has important theoretical and practical significance.

High-speed digital mobile communication is affected by frequency selective fading caused by multipath with various time delays [14]. Inter-symbol interference caused by frequency selective fading affects the correct demodulation of the demodulator, thereby limiting the maximum transmission data rate. In order to combat multipath fading, techniques such as diversity and equalization can be employed. The multipath separation technique is based on spread spectrum communication. Using the good autocorrelation property of the Pseudo-Noise Sequences, the receiver separates the multipath signal by correlating the multipath signal with multiple correlators [15,16]. However, the spread of the signal spectrum reduces the spectrum utilization and the demodulator complexity is high. In [17], a multipath separation method based on the EBPSK modulation system is proposed. This scheme is only applicable to binary EBPSK modulation, and there are fewer scenarios available. When sub-path information is used to participate in the decision, the multipath separation scheme based on EBPSK modulation introduces all sub-path noise. In order to further improve the frequency band utilization, it is very important to study the anti-fading of multi-ary modulation technology in efficient modulation. In this paper, based on the waveform characteristics of MPPSK modulation, a multipath separation method based on MPPSK modulation is proposed and the multipath separation conditions are given. The modulation parameters are adjusted by the multipath delay value to achieve the separation of the multipath and the main path, thereby reducing the inter-symbol interference of the multipath signal to the main path signal and improving the anti-multipath interference ability of the signal. Compared with the scheme of separating multipath by signal spread spectrum, this scheme reduces the spectrum overhead. Compared with the multipath separation scheme proposed in [17] for binary ultra-narrowband signals, the multipath separation method proposed in this paper is applicable to the M-ary MPPSK signals with higher transmission rate. By using the normalized symbol joint decision proposed in this paper, the receiver reduces the introduced noise while utilizing multipath information, thereby reducing the bit error rate of the system.

The rest of this paper is organized as follows. Section 2 introduces the channel model, MPPSK modulation and demodulation scheme. Section 3 introduces multipath delay estimation, modulation parameter design and normalized symbol joint decision. Section 4 presents the simulation results. Finally, Section 5 concludes the paper.

## 2. System Description

### 2.1. Mppsk Modulation

**Figure 1:**
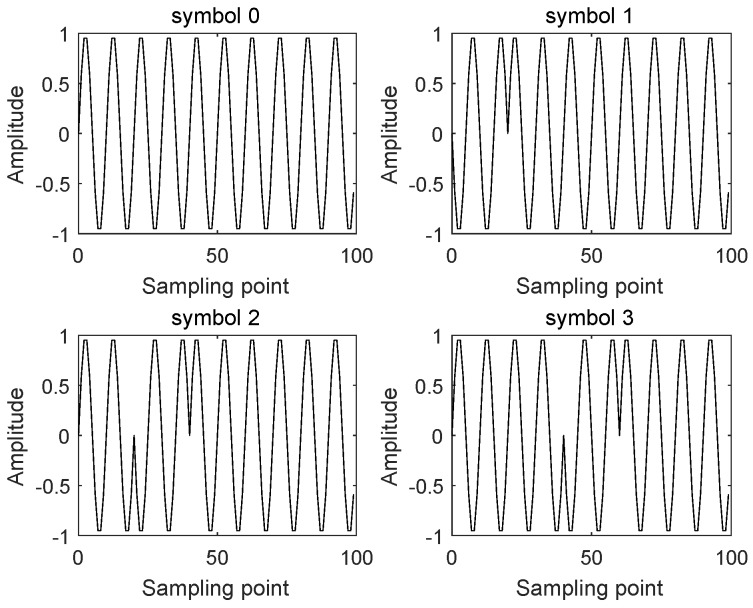
The MPPSK Waveforms.

MPPSK is a high spectral efficiency technique and a member of the sine-like modulation family [18]. MPPSK modulation is a modulation method extended from EBPSK to multi-ary modulation, which uses M-ary symbol to control the phase hopping position of sinusoidal carrier in each symbol period. Since MPPSK makes full use of the position change of the phase jump in the symbol, the information transmission rate is increased by $\log_{2}M$ times under the same bandwidth condition, thereby further improving the spectrum utilization rate. The expression of MPPSK modulation in a symbol period [0,NT] is given by

(1) $$f_{k}\left(t\right)=\left\{\begin{array}{c} \;\;\;Asin2\pi f_{c}t\;\;\;\;0\leq t<NT,k=0\\ \left\{\begin{array}{cc} Asin2\pi f_{c}t & 0\leq t<(k-1)KT\\ Bsin(2\pi f_{c}t+\theta ) & \left(k-1\right)KT<t<\left(k-r_{g}\right)KT,1\leq k\leq M-1\\ Asin2\pi f_{c}t & (k-r_{g})KT\leq t<NT \end{array}\right. \end{array}\right.$$

where *A* is the carrier amplitude of the symbol “0” and the non-zero symbol non-phase modulation interval, *B* is the carrier amplitude of the non-zero symbol phase modulation interval, k=0,1,2,…,M−1 is the actual transmitted symbol with M≥2 values, $T=\left(\frac{2\pi }{w_{c}}\right)$ is the carrier period, *N* is the number of carrier cycles in the symbol period, $T_{s}=NT$ is the symbol period, $(1-r_{g})K$ is the number of carrier cycles that the phase jump continues, 0<θ≤π is the angle of the phase jump, and $r_{g}\left(0\leq r_{g}<1\right)$ is the Guard Interval factor. In order to meet the requirements of different application indicators, different modulation signal bandwidths, transmission rates, and system demodulation performance can be obtained by adjusting the values of modulation parameters A,B,M,N,K,θ and $r_{g}$. The MPPSK signal in the specific case $A=B=1,r_{g}=0,N=10,K=2,\theta =\pi$ and M=4 are depicted in Figure 1.

### 2.2. Mppsk Demodulation

Special impacting filter is a special class of infinite impulse response (IIR) filters [19]. In very narrow passbands, Special impacting filter (SIF) has notch and select frequency characteristics. While filtering out noise, SIF can convert the phase jump of the MPPSK modulated signal into an amplitude impacting. Therefore, the modulation symbol can be demodulated by detecting the position of the impact at the demodulation terminal, which greatly simplifies the demodulation difficulty of the MPPSK system. The transfer function of SIF is given by

(2) $$H\left(z\right)=\frac{\sum_{i=0}^{L}b\left(i\right)z^{-i}}{1+\sum_{i=1}^{J}a\left(i\right)z^{-i}}$$

This paper takes the filter having two conjugate poles and one zero point, where

$$b_{0}=b_{2}=1;$$

$$b_{1}=-1.9021496572560159,$$

$$a_{1}=-3.6512241163814698,$$

$$a_{2}=5.1727286626648894,$$

$$a_{3}=-3.3577825365961242,$$

$$a_{4}=0.84572301542400019.$$

**Figure 2:**
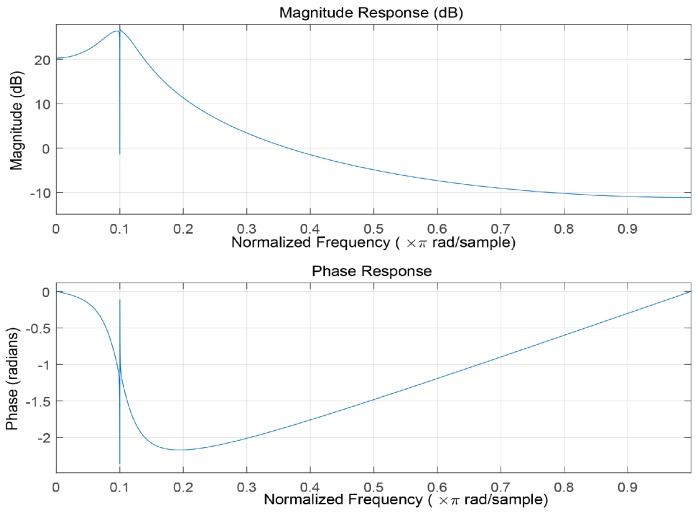
The MPPSK Waveforms.

The designed filter is suitable for MPPSK signal whose sampling frequency is 20 times of carrier frequency. Amplitude-frequency response and phase-frequency response of the SIF are shown in Figure 2.

**Figure 3:**
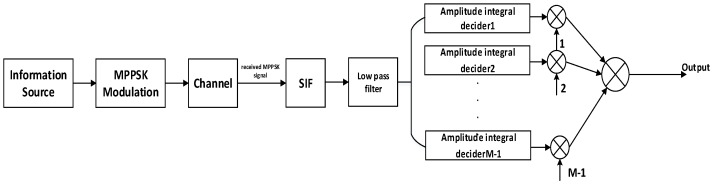
MPPSK system structure.

In this paper, the demodulation method based on amplitude integral decision is adopted. MPPSK system structure is shown in Figure 3.

**Figure 4:**
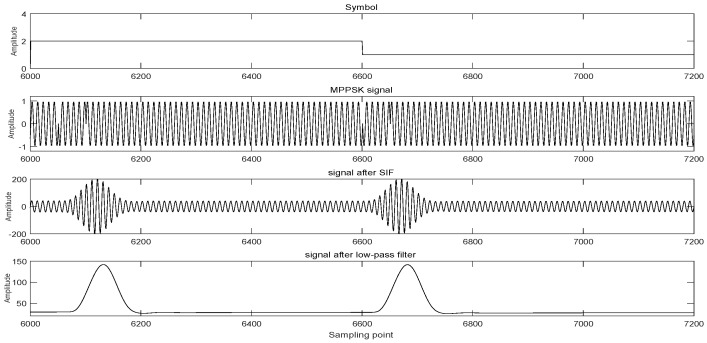
Waveform of MPPSK system.

The received MPPSK signal can transform phase jump into amplitude impact after passing through the impacting filter, and the envelope of the signal can be extracted after passing through the low pass filter for decision. The envelopes of M−1 phase hopping positions are sampled and integrated over the symbol period to find the maximum value of the integral and the associated slot. If the maximum is below the threshold level, the decoder outputs a zero value. Otherwise, the decoder allocates the symbols represented by the time slots corresponding to the largest integrated value [18]. The threshold level of the symbol is the average of the integral value at the phase jump of the symbol and the integral value at the corresponding position of the 0 symbol. The waveform of MPPSK system is shown in Figure 4.

### 2.3. High Frequency Multipath Channel

**Figure 5:**
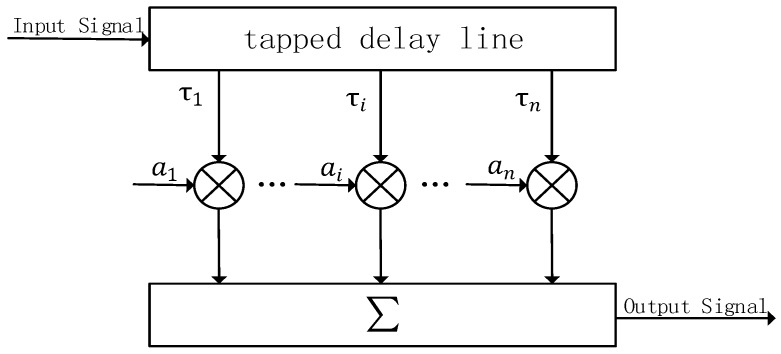
Channel Model.

HF channels are nonstationary in both frequency and time, but if consideration is restricted to band-limited channels (for example, 10 kHz) and sufficiently short times (for example, 10 min), most channels are nearly stationary and can be adequately represented by a stationary model [20]. The channel model is shown in Figure 5.

Where *n* is the number of paths, $\tau_{n}$ is the delay of each path, and $a_{n}$ is the gain of each path. The input signal is subjected to a finite order delay. Each order of the signal has an appropriate order gain to amplitude and phase modulate it. The modulated signal is summed at the output [20]. In this paper, when simulating the multipath effect, the multipath is regarded as the waveform of the main path delay. The received signal can be represented by superimposing the main path signal and the main path signal delayed for a certain time. It is assumed that the received expression of the transmitted MPPSK modulated signal is ag(t). After transmission over a multipath channel, the received signal is given by

(3) $$r\left(t\right)=\sum_{i=1}^{n}a_{i}g\left(t-\tau_{i}\right)$$

where $a_{i}$ is the amplitude fading caused by the gain of each path channel, and $\tau_{i}$ is the delay of each path relative to the main path.

## 3. The Scheme of Multipath Delay Estimation and Normalized Symbol Joint Decision

To reduce the interference of multipath to the signal, it is necessary to estimate the delay value of the multipath, and then design the modulation parameters of the signal to achieve the purpose of separating the multipath. At the demodulator end, not only the multipath information is used for the decision, but also the noise introduced by the multipath information is reduced, so that the demodulation performance can be improved.

### 3.1. Multipath Delay Estimation

Generalized cross-correlation (GCC) is one of the conventional methods for finding the time differences but it requires a priori statistics of the received signals in order to obtain accurate delay estimates [21]. In practical applications, these priori knowledge are often difficult to obtain or incomplete, which will affect the actual performance of this methods. On the other hand, due to the possible relative motion between the transmitter and the receiver, the time delay will change over time. These two problems will cause the GCC method to estimate the delay inaccuracy.

**Figure 6:**
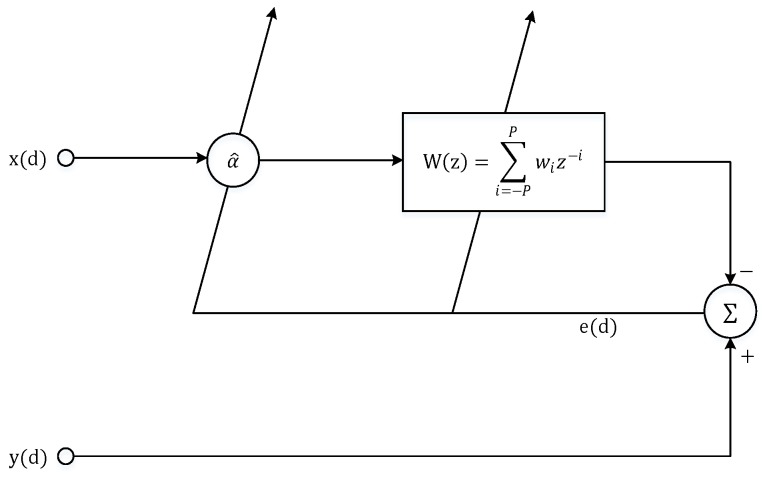
Block diagram of LMSTDE.

Compared to the above method, the least mean square time delay estimation(LMSTDE) method does not rely on statistical prior knowledge of the input signal and noise, and it can constantly adjust its parameters during the iterative process. In addition, the adaptive filter in the LMSTDE method is simple in design, low in computational complexity, and easy to implement. The block diagram of LMSTDE is shown in Figure 6 with $\hat{a}$ fixed at unity [22].

Where x(d) and y(d) are the two signals received by the receiver. The basic idea of LMSTDE method is to estimate the delay through an adaptive noncausal FIR filter in the form of $W\left(z\right)=\sum_{i=-P}^{P}w_{i}z^{-i}$. The delay value found with the least mean square error compared with the reference signal is the time delay estimate. The filter weight vector coefficients that are iterated according to the LMS algorithm are given by

(4) $$w_{i}\left(d+1\right)=w_{i}\left(d\right)+\mu_{w}e\left(d\right)x\left(d-i\right)$$

where the error $e\left(d\right)=y\left(d\right)-\sum_{i=-P}^{P}w_{i}\left(d\right)x\left(d-i\right)$, $\mu_{w}$ is a positive scalar to control the convergence rate and stability of the iteration. When the adaptive filter converges, a time delay estimate can be obtained from the weight vector peak coordinates of the adaptive filter. The delay ${\hat{D}}_{w}\left(d\right)$ is given by [23]

(5) $${\hat{D}}_{w}\left(d\right)=arg\max_{t}\left\{\sum_{i=-P}^{P}w_{i}\left(d\right)sinc\left(t-i\right)\right\}$$

where $sinc\left(\cdot \right)=\frac{sin\pi (\cdot )}{\pi (\cdot )}$.

### 3.2. Modulation Parameter Setting

Assume that the delay of the sub-path relative to the main path is Δτ. First, the integer part value *m* of $\frac{\Delta \tau }{T}$ is obtained, and *m* represents the number of symbol periods in which the sub-path is delayed from the main path. Then the parameter Δτ-mT is calculated, which is a specific position of sub-path in the (M+1)-th symbol period of main path. In order to separate the main path and the sub path signal, the condition is that the phase jump of the sub path after the delay cannot overlap with the phase jump of the main path. In this paper, taking only two paths of multipath as an example, according to the waveform characteristics of the MPPSK signal, the separation conditions of the main path and the sub path can be obtained.

1. When $0<\Delta \tau -mT<(M-1-r_{g})KT$, the main path delays m symbol periods to form a sub-path waveform whose waveform will fall within the modulation segment $\left(0-\left(M-1-r_{g}\right)KT\right)$ in the symbol period, so the sub-path will be aliased with the main path waveform.
2. When $\left(M-1-r_{g}\right)KT\leq \Delta \tau -mT<NT-\left(M-1-r_{g}\right)KT$, the main path delays m symbol periods to form a sub-path waveform, and the waveform will fall outside the symbol modulation period $\left(0-\left(M-1-r_{g}\right)KT\right)$ in the symbol period, so multipath does not affect the main path waveform. In order for this condition to be true, it is also necessary to satisfy $2(M-1-r_{g})K<N$.

Therefore, in order to satisfy this condition, after the carrier frequency of the transmitted signal is fixed, it is necessary to select the parameters *K* and *N* of the MPPSK pulse modulation signal according to the delay to satisfy the separation of the main paths and sub-paths.

### 3.3. Normalized Symbol Joint Decision

The premise of symbol joint decision is to estimate the multipath delay and then the separation of multipath. The key is to make the symbol information contained in the sub-path participate in the decision, that is, the sampling integral decision value of the current symbol and the sampling integral decision value including the current symbol in the multipath are used as the decision basis [17]. The symbol joint decision also introduces the noise of the sub-path when using the sub-path symbol information to participate in the decision, which causes the bit error rate not to reach the minimum level. Therefore, this paper proposes a normalized symbol joint decision method. After the signal is transmitted through multipath channel, the receiving expression is given by Equation (3). After obtaining the relative delay of each path, the delay value of the i-th path is eliminated and the waveform with no delay for each path is obtained. Then, each path signal is normalized and added to the main path, and the noise introduced by the sub-path is reduced while using the symbol information included in the sub-path, thereby improving the signal-to-noise ratio and reducing the bit error rate. The added signal is given by

(6) $$r\left(t\right)=\sum_{i=1}^{n}\frac{a_{i}}{a_{1}}a_{i}g\left(t\right)$$

where $a_{1}$ is the path gain of the main path and $\frac{a_{i}}{a_{1}}$ is the value obtained by normalizing the path gain of the i-th path with the main path gain. It is assumed that when the signal is transmitted, the energy of each symbol is $E_{s}$, and the amplitude gain of the i-th path is $a_{i}$, so the amplitude of the equivalent baseband signal on the i-th path is $a_{i}\sqrt{E_{s}}$, and the symbol energy is ${a_{i}}^{2}E_{s}$. Assuming that the noise power spectral density is $N_{0}$, the signal-to-noise ratio on the *i*-th path is given by

(7) $$\gamma_{i}=\frac{{a_{i}}^{2}E_{s}}{N_{0}}$$

when the received signals are combined, the weighting coefficient of each path is $x_{i}$, then the amplitude of the equivalent baseband signal of the i-th path after weighting is $x_{i}a_{i}\sqrt{E_{s}}$, and the amplitude of the combined signal is given by

(8) $$A=\sum_{i=1}^{n}x_{i}a_{i}\sqrt{E_{s}}$$

The signal-to-noise ratio of the combined signal is given by

(9) $$\begin{array}{c} \gamma \left(x_{1},x_{2},\cdots ,x_{n}\right)=\frac{{\left(\sum_{i=1}^{n}x_{i}a_{i}\sqrt{E_{s}}\right)}^{2}}{\sum_{i=1}^{n}{x_{i}}^{2}N_{0}}\\ =\frac{E_{s}}{N_{0}}\frac{{\left(\sum_{i=1}^{n}x_{i}a_{i}\right)}^{2}}{\sum_{i=1}^{n}{x_{i}}^{2}} \end{array}$$

1. When the receiver adopts the symbol joint decision scheme, the weighting coefficient of each path is 1, that is, $x_{i}=1\left(i=1,2,\cdots ,n\right)$. The signal-to-noise ratio of the combined signal is given by
  (10) $$\gamma_{S}\left(x_{1},x_{2},\cdots ,x_{n}\right)=\frac{E_{s}}{N_{0}}\frac{{\left(\sum_{i=1}^{n}a_{i}\right)}^{2}}{\sum_{i=1}^{n}1^{2}}$$
2. When the receiver adopts the normalized symbol joint decision scheme, the weighting coefficients of the i-th path is $\frac{a_{i}}{a_{1}}$, that is, $x_{i}=\frac{a_{i}}{a_{1}}$. The signal-to-noise ratio of the combined signal is given by
  (11) $$\gamma_{N}\left(x_{1},x_{2},\cdots ,x_{n}\right)=\frac{E_{s}}{N_{0}}\frac{{\left(\sum_{i=1}^{n}\frac{a_{i}^{2}}{a_{1}}\right)}^{2}}{\sum_{i=1}^{n}{\left(\frac{a_{i}}{a_{1}}\right)}^{2}}$$

The calculation shows that $\gamma_{N}\left(x_{1},x_{2},\cdots ,x_{n}\right)$ is larger than $\gamma_{S}\left(x_{1},x_{2},\cdots ,x_{n}\right)$, that is, the normalized symbol joint decision scheme improves the signal-to-noise ratio and reduces the bit error rate compared with the symbol joint decision scheme. The proof is shown in Appendix A.

## 4. Simulation and Numerical Results

In this section, time delay and joint decision are simulated for signals with modulation parameters K=5,N=60 and $f_{c}=455$ KHZ. Table 1 gives the estimated delays for using the LMSTDE scheme with a delay of 1 ms. Table 2 gives an estimate of the multipath delay by using an autocorrelation scheme. The comparison results of bandwidth between modulation parameter schemes and spread spectrum schemes are given in Table 3. In addition, the normalized symbol decision method is compared with the the current mainstream method by taking the signal-to-noise ratio and the sub-path gain as variables.

### 4.1. Time Delay Estimation

**Figure 7:**
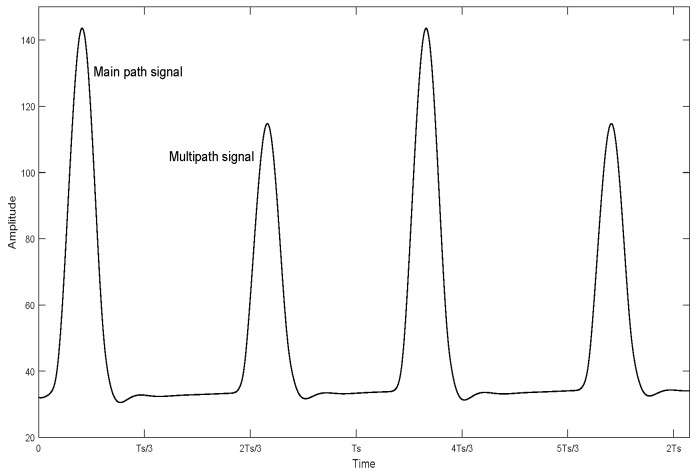
Waveform of two symbol periods after the received signal is demodulated.

According to the requirements of modulation parameter setting, the modulation parameters selected in this paper are $K=5,N=60,f_{c}=455$ KHZ and Δτ=1 ms, and there are only two paths of main path and sub-path. The delay range is calculated to get m value (m=7), and the Δτ−mT conforms to the second case of parameter setting in Section 3, that is, the delay waveform of the previous seventh symbol is included in the current symbol period, and the delay waveform of the current symbol is included in the seventh symbol period later. When the main path and the sub-path can be separated, the signal waveform of the received signal in two symbol periods after demodulation is shown in Figure 7.

It can be seen from Figure 7 that when the MPPSK modulated signal passes through the multipath channel, two peaks appear in one symbol period, one of which is the decision peak of the current symbol, and the other is the multipath peak of the previous seventh symbol delay to the current symbol period. When the signal-to-noise ratio takes different values, the signal modulation parameter multipath gain is modified, and the multipath delay is estimated using the LMSTDE method. The delay value of the sub-path relative to the main path is given in Table 1.

As can be seen from Table 1, when the multipath gain is modified, the accuracy of time delay estimation using LMSTDE method is not significantly affected. Therefore, the LMSTDE method can be used to estimate the time delay of MPPSK multipath signals.

The traditional multipath separation technique spreads the signal through a Pseudo-Noise Sequences of a specific design, and the receiver performs correlation operations to separate the multipath. Since the Pseudo-Noise Sequences has a sharp autocorrelation property, the receiver sends the received multipath signal to a plurality of correlators, and each path signal can be separated. The premise of multipath separation is multipath delay estimation. Therefore, after spreading the MPPSK signal spectrum, a m-sequence known to the receiver is inserted in front of the symbol to be transmitted. When the receiver correlates the received multipath signal with the known m-sequence, the multipath delay can be determined by the time interval between the peaks of the correlation function graph. With the same parameters as above, the multipath delay is estimated by using autocorrelation method under different signal-to-noise ratios, different multipath gains and different m-sequences. When using the autocorrelation method, the delay of the sub-path relative to the main path is shown in Table 2.

Table 2 shows that since m-sequence has good autocorrelation characteristics, the time delay of MPPSK signal after the spread spectrum can be estimated by autocorrelation method, so that multipath separation can be achieved in the receiver. To measure the bandwidth of the two multipath separation schemes mentioned above, one is to adjust the modulation parameters to separate the multipath, the other is to separate the multipath by using the correlation reception method after the spread spectrum of the signal. When the spectrum of 4PPSK signal is spread, four quaternary symbols are corresponding to four different binary sequences, and then the binary sequence is modulated by the m-sequence, and finally the signal that needs to be transmitted is obtained by 4PPSK modulation. The corresponding relationship is 0 corresponding to 00, 1 corresponding to 01, 2 corresponding to 11, and 3 corresponding to 10. The −40 dB bandwidth of the two separated multipath schemes is shown in Table 3.

As shown in Table 3, although both schemes can separate multipaths, the spread spectrum scheme has wider bandwidth than the modulation parameter adjustment scheme. The scheme of adjusting the modulation parameters can separate the multipath and eliminate the inter-symbol interference only by requiring appropriate modulation parameters, so the separation method is relatively simple. In the case of transmitting the same information, adjusting the modulation parameter scheme saves spectrum resources and improves frequency band utilization.

### 4.2. Bit Error Rate of Normalized Symbol Joint Decision

Under the condition that the sub-path can be separated from the main path, the current decision of the symbol can be understood as a joint decision between the sampled integrated value of the normalized sub-path and the sampled integrated value of the main path. This can be understood as the introduction of “encoding“ in the transmitter. The coding mode is to encode the information of the current symbol into the following seventh symbol without affecting the information representation of the following seventh symbol, thereby ensuring that the current symbol demodulation performance is improved without lowering the symbol rate.

**Figure 8:**
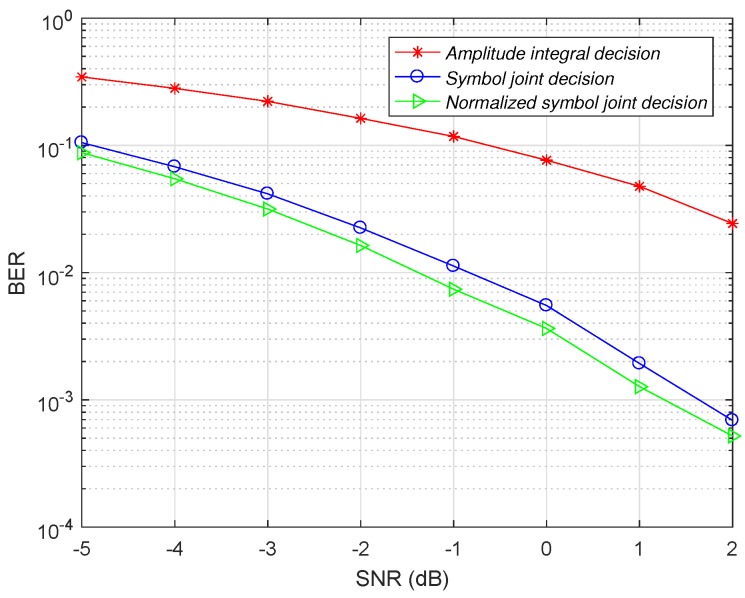
Bit error rate comparison of three decision schemes.

The modulation parameters and channel conditions are the same as the Time delay estimation section. In the case where the main path gain is 0.8 and the sub path gain is 0.5, the demodulation performance of the amplitude integral decision, the symbol joint decision and the normalized symbol joint decision is compared. The comparison result is shown in Figure 8.

**Figure 9:**
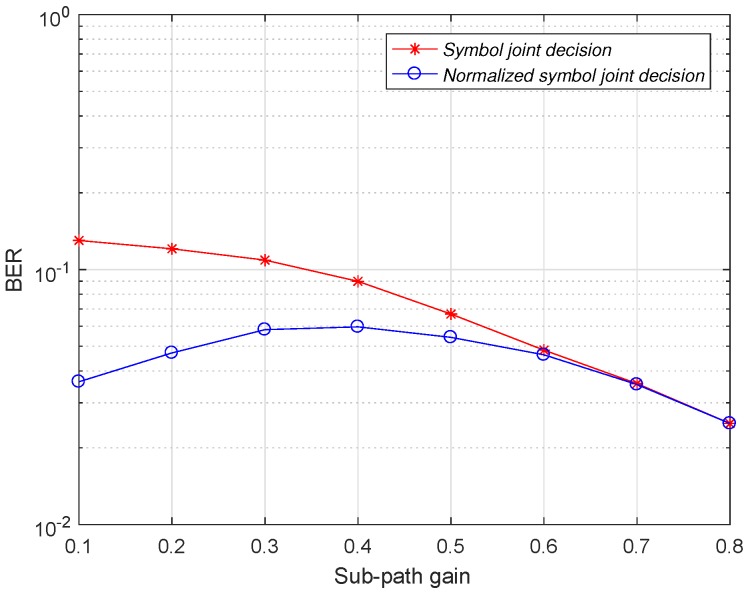
Bit error rate comparison of normalized symbol joint decision and symbol joint decision after fixed main path gain.

As shown in Figure 8, the normalized symbol joint decision reduces the bit error rate of the demodulator compared to the amplitude integral decision and the symbol joint decision. In the case of low signal-to-noise ratio conditions, the normalized symbol joint decision is improved compared with the symbol joint decision in terms of bit error rate. As the signal-to-noise ratio increases, the noise signal gradually decreases. Therefore, the weakening effect of the normalized symbol joint decision on the noise is gradually reduced, and the performance improvement of the normalized symbol joint decision is gradually reduced relative to the joint decision of the symbol. When the signal-to-noise ratio is −4 dB, the channel gain of the main path is fixed at 0.8. The effect of the change in the sub-path gain on the demodulation performance of the symbol joint decision and the normalized symbol joint decision is shown in Figure 9.

**Figure 10:**
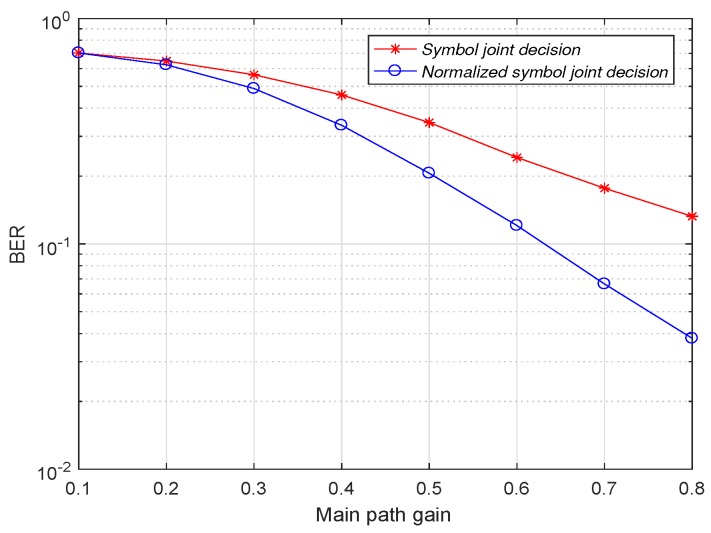
Bit error rate comparison of normalized symbol joint decision and symbol joint decision after fixed sub-path gain.

As shown in Figure 9, after fixing the main path gain, the demodulator uses the normalized symbol joint decision with a lower bit error rate than the use of the symbol joint decision at different sub-path gains. When the sub-path gain is 0.1, the normalized symbol joint decision has the highest improvement in demodulation performance relative to the symbol joint decision, because the normalized symbol joint decision not only utilizes the symbol information of the sub-path to participate in the decision, but also minimizes the introduced noise. As the gain of the sub-path increases, the normalized value of the sub-path gain to the main path gain becomes closer to 1, that is, the normalized symbol joint decision introduces more and more noise when using the sub-path symbol information. This leads to a gradual decrease in the advantages of the normalized symbol joint decision and the symbol joint decision. Finally, when the main path gain and the sub path gain are the same, the demodulation performance of the two decision modes is the same. When the signal-to-noise ratio is −4 dB, the channel gain of the sub-path is fixed at 0.1. The effect of the change in the main path gain on the demodulation performance of the symbol joint decision and the normalized symbol joint decision is shown in Figure 10.

As shown in Figure 10, the conclusion is the same as when the main path gain is fixed. Compared with the sub-path gain, the bit error rate is the smallest when the main path gain is maximum.

**Figure 11:**
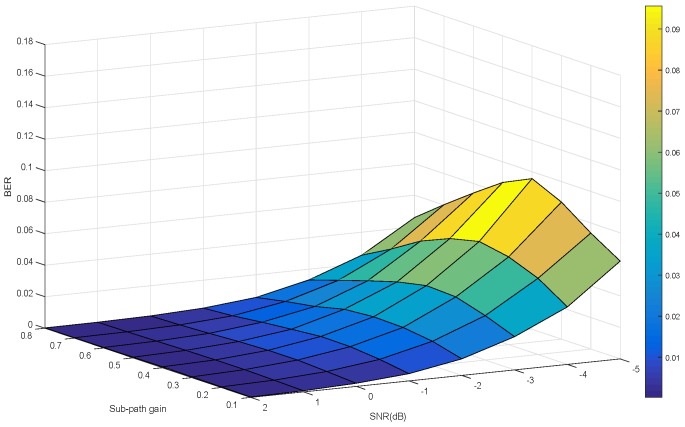
BER for normalized symbol joint decision as a function of SNR and sub-path gain.

Figure 11 shows that when the demodulator uses the normalized symbol joint decision, the bit error rate is a function of the signal to noise ratio and the sub-path gain.

**Figure 12:**
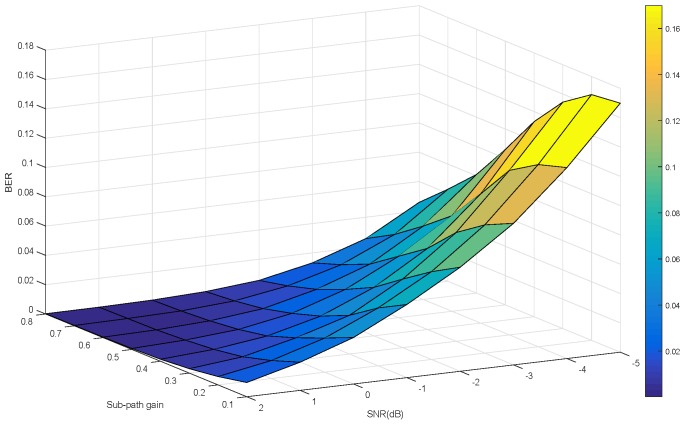
BER for symbol joint decision as a function of SNR and sub-path gain.

Figure 12 indicates that the BER is a function of SNR and sub-path gain as independent variables when the demodulator uses the symbol joint decision.

Comparing Figure 11 with Figure 12, considering the influence of signal-to-noise ratio and multipath gain, it can be seen that the normalized symbol joint decision proposed in this paper is superior to the symbol joint decision in reducing the bit error rate.

## 5. Conclusions

In this paper, the effectiveness of the least mean square time delay estimation method for the estimation of MPPSK-modulated signal delay in a multipath environment is verified by experiments. By analyzing the waveform characteristics of the MPPSK signal, the conditions of multipath separation are given. Using the obtained delay estimation value, the modulation parameters of the system can be selected to achieve the separation of the main path and the multipath. Compared with the multipath separation achieved by the spread spectrum scheme, the multipath separation method proposed by adjusting the MPPSK modulation parameters in this paper has a narrower bandwidth and higher spectrum utilization. The complexity of the whole system is low, and the demodulator reduces the introduced noise while using the multipath information to participate in the decision. The demodulation performance is improved compared with the existing method. The results show that the normalized symbol joint decision method can effectively combat multipath. As the sub-path gain gradually decreases, the advantage of the normalized symbol joint decision becomes more and more obvious. Compared with the previous methods, the method reduces the error rate of the demodulator. Future work will probably study the performance of MPPSK modulation in fading, strong interference environments and the optimization of the demodulator.

## Figures and Tables

**Table 1:** LMSTDE Delay Estimation with Multipath Gain Change.

SNR (dB)	Parameter K	Multipath Gain	Delay (s)
−5	5	0.1	$-0.966\times 10^{-3}$
	5	0.5	$-0.949\times 10^{-3}$
	5	0.8	$-0.965\times 10^{-3}$
−4	5	0.1	$-0.992\times 10^{-3}$
	5	0.5	$-0.956\times 10^{-3}$
	5	0.8	$-1.034\times 10^{-3}$
−3	5	0.1	$-0.983\times 10^{-3}$
	5	0.5	$-0.937\times 10^{-3}$
	5	0.8	$-0.964\times 10^{-3}$
−2	5	0.1	$-0.987\times 10^{-3}$
	5	0.5	$-0.993\times 10^{-3}$
	5	0.8	$-0.955\times 10^{-3}$
−1	5	0.1	$-1.026\times 10^{-3}$
	5	0.5	$-0.961\times 10^{-3}$
	5	0.8	$-0.969\times 10^{-3}$
0	5	0.1	$-0.958\times 10^{-3}$
	5	0.5	$-0.944\times 10^{-3}$
	5	0.8	$-0.937\times 10^{-3}$
1	5	0.1	$-0.956\times 10^{-3}$
	5	0.5	$-0.926\times 10^{-3}$
	5	0.8	$-1.024\times 10^{-3}$
2	5	0.1	$-0.966\times 10^{-3}$
	5	0.5	$-1.016\times 10^{-3}$
	5	0.8	$-1.023\times 10^{-3}$
3	5	0.1	$-0.989\times 10^{-3}$
	5	0.5	$-1.017\times 10^{-3}$
	5	0.8	$-0.980\times 10^{-3}$

**Table 2:** Autocorrelation Scheme Delay Estimation with Multipath Gain Change.

SNR (dB)	Period of m-Sequence	Multipath Gain	Delay (s)
−5	7	0.1	$-0.793\times 10^{-3}$
		0.5	$-1.198\times 10^{-3}$
		0.8	$-0.820\times 10^{-3}$
	15	0.1	$-0.763\times 10^{-3}$
		0.5	$-0.736\times 10^{-3}$
		0.8	$-0.868\times 10^{-3}$
	31	0.1	$-0.707\times 10^{-3}$
		0.5	$-0.809\times 10^{-3}$
		0.8	$-0.872\times 10^{-3}$
−3	7	0.1	$-0.737\times 10^{-3}$
		0.5	$-0.776\times 10^{-3}$
		0.8	$-0.812\times 10^{-3}$
	15	0.1	$-0.718\times 10^{-3}$
		0.5	$-0.859\times 10^{-3}$
		0.8	$-0.877\times 10^{-3}$
	31	0.1	$-0.801\times 10^{-3}$
		0.5	$-0.937\times 10^{-3}$
		0.8	$-0.937\times 10^{-3}$
−1	7	0.1	$-0.778\times 10^{-3}$
		0.5	$-0.850\times 10^{-3}$
		0.8	$-0.868\times 10^{-3}$
	15	0.1	$-0.737\times 10^{-3}$
		0.5	$-0.851\times 10^{-3}$
		0.8	$-0.833\times 10^{-3}$
	31	0.1	$-0.889\times 10^{-3}$
		0.5	$-1.127\times 10^{-3}$
		0.8	$-1.063\times 10^{-3}$
1	7	0.1	$-0.831\times 10^{-3}$
		0.5	$-0.849\times 10^{-3}$
		0.8	$-0.886\times 10^{-3}$
	15	0.1	$-0.859\times 10^{-3}$
		0.5	$-0.886\times 10^{-3}$
		0.8	$-1.131\times 10^{-3}$
	31	0.1	$-0.890\times 10^{-3}$
		0.5	$-1.068\times 10^{-3}$
		0.8	$-0.941\times 10^{-3}$
3	7	0.1	$-0.906\times 10^{-3}$
		0.5	$-0.906\times 10^{-3}$
		0.8	$-0.982\times 10^{-3}$
	15	0.1	$-0.877\times 10^{-3}$
		0.5	$-0.903\times 10^{-3}$
		0.8	$-1.017\times 10^{-3}$
	31	0.1	$-0.903\times 10^{-3}$
		0.5	$-0.873\times 10^{-3}$
		0.8	$-1.059\times 10^{-3}$

**Table 3:** −40 dB Bandwidth of The Two Separated Multipath Schemes.

Period of m-Sequence	Adjust Modulation Parameter Scheme	Spread Spectrum Scheme
7	0.295 MHZ	7.001 MHZ
15	0.295 MHZ	11.831 MHZ
31	0.295 MHZ	20.171 MHZ

## Abbreviations

The following abbreviations are used in this manuscript:

LPWAN	Low-Power Wide-Area Network
IoT	Internet of Things
UNB	Ultra-Narrowband
MPPSK	M-ary Position Phase Shift Keying
EBPSK	Extend Binary Phase Shift Keying
DIF	Digital Impacting Filter
IIR	Infinite Impulse Response
SIF	Special Impacting Filter
HF	High Frequency
GCC	Generalized cross-correlation
LMSTDE	Least Mean Square Time Delay Estimation

## Appendix A

This proof is based on mathematical induction. If the receiver has a larger signal-to-noise ratio using the normalized symbol joint decision scheme than using the symbol joint decision scheme, the comparison result is given by

(A1) $$\begin{array}{c} \gamma_{N}\left(x_{1},x_{2},\cdots ,x_{n}\right)>\gamma_{S}\left(x_{1},x_{2},\cdots ,x_{n}\right) \end{array}$$

among them,

(A2) $$\begin{array}{c} \begin{array}{cc} \gamma_{S}\left(x_{1},x_{2},\cdots ,x_{n}\right) & =\frac{E_{s}}{N_{0}}\frac{{\left(\sum_{i=1}^{n}a_{i}\right)}^{2}}{\sum_{i=1}^{n}1^{2}}\\ & =\frac{E_{s}}{N_{0}}\frac{{\left(\sum_{i=1}^{n}a_{i}\right)}^{2}}{n} \end{array} \end{array}$$

(A3) $$\begin{array}{c} \begin{array}{cc} \gamma_{N}\left(x_{1},x_{2},\cdots ,x_{n}\right) & =\frac{E_{s}}{N_{0}}\frac{{\left(\sum_{i=1}^{n}\frac{a_{i}^{2}}{a_{1}}\right)}^{2}}{\sum_{i=1}^{n}{\left(\frac{a_{i}}{a_{1}}\right)}^{2}}\\ & =\frac{E_{s}}{N_{0}}\frac{{\left(\sum_{i=1}^{n}a_{i}^{2}\right)}^{2}{\left(\frac{1}{a_{1}}\right)}^{2}}{\sum_{i=1}^{n}{\left(a_{i}\right)}^{2}{\left(\frac{1}{a_{1}}\right)}^{2}}\\ & =\frac{E_{s}}{N_{0}}\frac{{\left(\sum_{i=1}^{n}a_{i}^{2}\right)}^{2}}{\sum_{i=1}^{n}{\left(a_{i}\right)}^{2}}\\ & =\frac{E_{s}}{N_{0}}\sum_{i=1}^{n}{\left(a_{i}\right)}^{2} \end{array} \end{array}$$

Since $a_{i}$ is the *i*-th path gain, $1\geq a_{1}>a_{2}>\cdots \cdots >a_{n}>0$ exists.

(A4) $$\begin{array}{cc} ∵\gamma_{N}\left(x_{1},x_{2},\cdots ,x_{n}\right) & >\gamma_{S}\left(x_{1},x_{2},\cdots ,x_{n}\right) \end{array}$$

(A5) $$\begin{array}{cc} ∴\frac{E_{s}}{N_{0}}\sum_{i=1}^{n}{\left(a_{i}\right)}^{2} & >\frac{E_{s}}{N_{0}}\frac{{\left(\sum_{i=1}^{n}a_{i}\right)}^{2}}{n} \end{array}$$

Since $\frac{E_{s}}{N_{0}}$ is greater than 0, the formula to be proved is given by

(A6) $$\begin{array}{cc} n\sum_{i=1}^{n}{\left(a_{i}\right)}^{2} & >{\left(\sum_{i=1}^{n}a_{i}\right)}^{2} \end{array}$$

when n=2,

(A7) $$\begin{array}{cc} ∵{\left(a_{1}+a_{2}\right)}^{2} & >0 \end{array}$$

(A8) $$\begin{array}{cc} ∴a_{1}^{2}+a_{2}^{2} & >2a_{1}a_{2} \end{array}$$

(A9) $$\begin{array}{cc} ∴2(a_{1}^{2}+a_{2}^{2}) & >a_{1}^{2}+a_{2}^{2}+2a_{1}a_{2} \end{array}$$

(A10) $$\begin{array}{cc} ∴2(a_{1}^{2}+a_{2}^{2}) & >{\left(a_{1}+a_{2}\right)}^{2} \end{array}$$

When n=m, the inequality is workable.

(A11) $$\begin{array}{c} m\left(a_{1}^{2}+a_{2}^{2}+\cdots +a_{m}^{2}\right)>{\left(a_{1}+a_{2}+\cdots +a_{m}\right)}^{2} \end{array}$$

when n=m+1,

(A12) $$\begin{array}{cc} {\left(a_{1}+a_{2}+\cdots +a_{m}+a_{m+1}\right)}^{2} & ={\left(a_{1}+a_{2}+\cdots +a_{m}\right)}^{2}+2(a_{1}+a_{2}+\cdots \\ & +a_{m})a_{m+1}+a_{m+1}^{2} \end{array}$$

(A13) $$∵m\left(a_{1}^{2}+a_{2}^{2}+\cdots +a_{m}^{2}\right)>{\left(a_{1}+a_{2}+\cdots +a_{m}\right)}^{2}$$

(A14) $$\begin{array}{cc} ∴(a_{1}+a_{2}+\cdots + & a_{m}{)}^{2}+2\left(a_{1}+a_{2}+\cdots +a_{m}\right)a_{m+1}+a_{m+1}^{2}<m(a_{1}^{2}+\\ & a_{2}^{2}+\cdots +a_{m}^{2})+a_{m+1}^{2}+2a_{1}a_{m+1}+\cdots +2a_{m}a_{m+1} \end{array}$$

(A15) $$\begin{array}{c} ∵m\left(a_{1}^{2}+a_{2}^{2}+\cdots +a_{m}^{2}\right)+a_{m+1}^{2}+2a_{1}a_{m+1}+\cdots +2a_{m}a_{m+1}<m(a_{1}^{2}+\\ a_{2}^{2}+\cdots +a_{m}^{2})+a_{m+1}^{2}+a_{1}^{2}+a_{2}^{2}+\cdots +a_{m}^{2}+ma_{m+1}^{2} \end{array}$$

(A16) $$\begin{array}{cc} ∵m\left(a_{1}^{2}+a_{2}^{2}+\cdots +a_{m}^{2}\right)+a_{m+1}^{2}+ & a_{1}^{2}+a_{2}^{2}+\cdots +a_{m}^{2}+ma_{m+1}^{2}=(m\\ & +1)\left(a_{1}^{2}+a_{2}^{2}+\cdots +a_{m}^{2}+a_{m+1}^{2}\right) \end{array}$$

(A17) $$\begin{array}{cc} ∴\left(m+1\right)\left(a_{1}^{2}+a_{2}^{2}+\cdots +a_{m}^{2}+a_{m+1}^{2}\right) & >{\left(a_{1}+a_{2}+\cdots +a_{m}+a_{m+1}\right)}^{2} \end{array}$$

To sum up, the following formula is true.

(A18) $$\begin{array}{cc} n\sum_{i=1}^{n}{\left(a_{i}\right)}^{2} & >{\left(\sum_{i=1}^{n}a_{i}\right)}^{2} \end{array}$$

Hence,

(A19) $$\begin{array}{cc} \gamma_{N}\left(x_{1},x_{2},\cdots ,x_{n}\right) & >\gamma_{S}\left(x_{1},x_{2},\cdots ,x_{n}\right) \end{array}$$
